# Silica-titania xerogel for solid phase spectrophotometric determination of salicylate and its derivatives in biological liquids and pharmaceuticals

**DOI:** 10.1186/s13065-015-0142-z

**Published:** 2015-11-25

**Authors:** Maria A. Morosanova, Elena I. Morosanova

**Affiliations:** Analytical Chemistry Division, Chemistry Department, Lomonosov Moscow State University, Moscow, Russia

**Keywords:** Silica-titania xerogels, Solid phase spectrophotometric determination, Salicylate, Acetylsalicylic acid, Salicylamide, Methylsalicylate, Human urine, Synthetic serum, Pharmaceuticals analysis

## Abstract

**Background:**

Salicylic acid and its derivatives are widely used drugs with potential toxicity. The main areas of salicylate derivatives determination are biological liquids and pharmaceuticals analysis.

**Results:**

Silica-titania xerogel has been used for solid phase spectrophotometric determination of various salicylate derivatives (salicylate, salicylamide, methylsalicylate). The reaction conditions influence on the interaction of salicylate derivatives with silica-titania xerogels has been investigated; the characteristics of titanium(IV)-salicylate derivatives complexes in solid phase have been described. The simple solid phase spectrophotometric procedures are based on the formation of xerogel incorporated titanium(IV) colored complexes with salicylate derivatives. A linear response has been observed in the following concentration ranges 0.1–5, 0.5–10 and 0.05-4.7 mM for salicylate, salicylamide, and methylsalicylate, respectively. The proposed procedures have been applied to the analysis of human urine, synthetic serum, and pharmaceuticals.

**Conclusions:**

The simple solid phase spectrophotometric procedures of salicylate derivatives determination based on the new sensor materials have been proposed for biological liquids and pharmaceuticals analysis.

**Graphical abstract:**
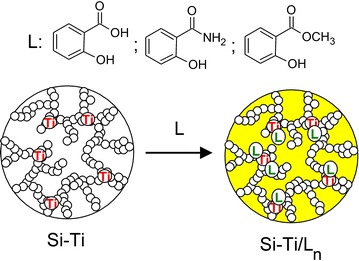
Complexation of titanium (IV), incorporated in silica-titania xerogels (Si-Ti), with salicylate derivatives (L) resulting in yellow-colored xerogels (Si-Ti/L_n_) has been proposed for salicylate derivatives determination in biological liquids and pharmaceuticals

## Background

Salicylic acid and its derivatives (acetylsalicylic acid, salicylamide, methylsalicylate) are widely used as anti-inflammatory, analgesic drugs [1]. Acetylsalicylic acid is the most commonly used salicylate derivative, it is used as analgesic, antipyretic, and also as an antiplatelet drug. Salicylamide is used as an analgesic and antipyretic in several combination products. It is necessary to control their presence in biological fluids due to the potential toxicity. Methylsalicylate is also used as an anti-inflammatory drug, but it is highly toxic if ingested and is only prescribed for external application. The drugs of this group have common pharmacological effect and all of them are toxic in high concentrations. A plasma level higher than 2.2 mM of salicylate is considered to be toxic [2], and the level higher than 4.3 mM is regarded as lethal [1]. The therapeutic range (0.5–1.5 mM of salicylate in plasma) is very close to the toxicity level.

Monitoring salicylates concentration in biological liquids is important for controlling the dose and frequency of salicylate derivative drug administration as all the salicylate derivatives mostly convert to salicylate in the organism. Accidental overdoses of salicylates are considered to be common in children. Salicylates are one of the toxicants that must be determined in serum and urine of patients of emergency department [3]. The allergenic capacity of salicylates also dictates the necessity of monitoring their levels in biological liquids. Another essential field for salicylate derivatives analysis is the pharmaceuticals quality control.

Various methods have been proposed for the determination of salicylic acid and its derivatives in biological liquids and pharmaceutical preparations: chromatography [4, 5], spectroscopic [6–9], electrochemical [10–15]. Many spectrophotometric and electrochemical methods of salicylate determination are based on its ability of forming complexes with metals: colored complex of salicylate with iron (III) is used in the classical method of salicylate determination (Trinder test) in biological samples [6]; complexing reaction with transition metal ions is employed in ion-selective electrodes construction [12–14].

The development of the simple methods of salicylate determination is a high-demand task, considering the wide use of salicylate derivatives in medical practice. The search for new sensor materials with an ability to form complexes with salicylates is a highly perspective goal.

Titanium(IV) forms colored complexes in weakly acidic solutions with some aliphatic and aromatic ligands. For example, salicylic acid and 5-chlorosalicylic acid are widely used for spectrophotometric determination of titanium(IV) [16].

In our previous works the ability of titanium(IV) embedded in silica-titania xerogel matrix to form complexes with ascorbic acid, polyphenols, dopamine, hydrogen peroxide, and fluoride ions was exploited to develop the solid phase spectrophotometric procedures for these substances determination [17–20].

The aim of the present work was to study the complex forming between the silica-titania xerogel and salicylate, salicylamide, or methylsalicylate and to choose the conditions of these salicylate derivatives and also acetylsalicylic acid determination in pharmaceuticals and salicylate determination in biological liquids.

## Results and discussions

### Interaction of silica-titania xerogel with salicylate derivatives

**Fig. 1:**
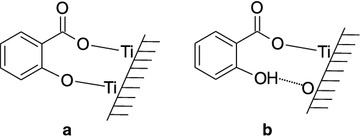
Two possible complexes of salicylate with titanium(IV) on the titanium dioxide surfaces [22]. **a** The complex of salicylate with two titanium(IV) ions, **b** the complex of salicylate with one titanium(IV) ion

The complexes of salicylate and titanium(IV) in solid phase are discussed in literature. The interaction of titanium dioxide surfaces with various complexing agents, including salicylate, is described. The important interaction which is involved in the titanium(IV) butoxyde–salicylate complex formation in mixed crystals is the hydrogen bonds formation [21]. It is different from the titanium(IV) butoxyde–catechol complexes, which are shown to rely mostly on van der Waals interactions. In [22] the hydrogen bonds are also shown to be important for salicylate-titanium complexes formation on the surface of solid titanium dioxide, as they are necessary for surface titanium(IV) to retain their normal coordination. However, the hydrogen bond is only formed if salicylate interacts with one titanium ion, and not when salicylate interacts with two titanium ions (the schemes of these two complexes are presented in Fig. 1). The complexes of titanium(IV) with salicylamide and methylsalicylate in solid phase have not been studied. As the described above materials have surfaces similar to those of our silica-titania xerogels the complexes may also be similar.

In the present work the silica-titania xerogel used for complex formation study was prepared using the sol–gel technology. Tetraethoxysilane and titanium(IV) tetraethoxyde were used as precursors. The xerogel with 12.5 % titanium(IV) tetraethoxyde content was chosen considering the micropore distribution analysis [20]. Then the optimal conditions for the complex forming reaction were investigated.

**Fig. 2:**
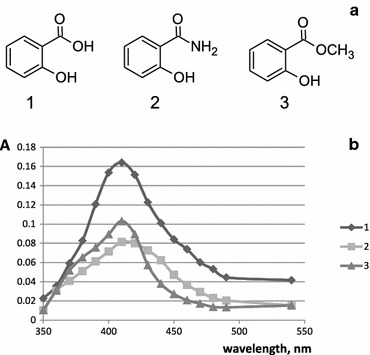
Absorption spectra of xerogels after reaction with salicylate derivatives (time of contact with salicylate derivatives is 15 min). **a** Chemical structures of salicylate derivatives and **b** absorption spectra of xerogels after reaction with salicylate derivatives 1 mM solutions. *1* sodium salicylate (pH 2.0), *2* salicylamide (pH 2.0), *3* methylsalicylate (pH 7.6)

The interaction of the silica-titania xerogel with salicylate, salicylamide, and methylsalicylate was studied in the present work. After the contact of the silica-titania xerogel with salicylate derivatives the xerogel’s color changed from white to pale yellow which signified the complex formation. The xerogels spectra after complex forming reaction with salicylate derivatives showed broad absorption bands at 390–420 nm the maxima being 410 nm (Fig. 2). When compared to the spectra of studied salicylate derivatives the xerogel spectra displayed significant bathochromic shift (70 nm for salicylate, 90 nm for salicylamide, 95 nm for methylsalicylate). In the following experiments colored xerogels absorbance was measured at 410 nm.

**Fig. 3:**
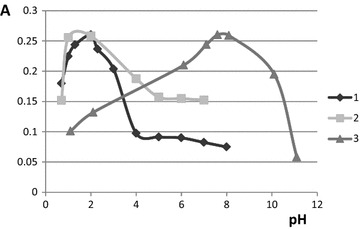
The dependence of silica-titania xerogels absorbance on pH after the contact with salicylate derivatives. λ 410 nm, time of contact with salicylate derivatives is 15 min. *1* 2 × 10^−3^ M sodium salicylate, *2* 3 × 10^−3^ M salicylamide, *3* 2.5 × 10^−3^ M methylsalicylate

The reaction conditions (pH of the solution and reaction time) influence on the complexing reaction was studied. The pH influence was studied in the range of 1.0-11.0 and the optimum was found out to vary for the different salicylate derivatives (Fig. 3). The maximal values of the xerogels absorbance were observed at pH 1.5–2.5 for salicylate and salicylamide and at pH 7.0–8.0 for methylsalicylate. In the case of salicylate and salicylamide the pH increase leads to the decrease of the formed complexes amount which results in the decrease of the absorbance value. These data correspond with the literature: the acidic media (pH < 2.5) has been reported to be optimal for the salicylate-titanium complexes formation on the surface of solid titanium dioxide [22]. The degradation of methylsalicylate in the acidic media was described in [4], as it was observed by liquid chromatographic method; such degradation could contribute to the pH optimum shifting for methylsalicylate. In the following experiments the following conditions were used for the reactions: pH 2.0 for salicylate and salicylamide and pH 7.6 for methylsalicylate.

**Fig. 4:**
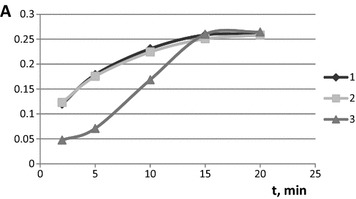
The dependence of silica-titania xerogels absorbance on the time of contact with salicylate derivatives. λ 410 nm. *1* 2 × 10^−3^ M sodium salicylate, pH 2.0, *2* 3∙× 10^−3^ M salicylamide, pH 2.0, *3* 2.5∙× 10^−3^ M methylsalicylate, pH 7.6

The effect of contact time with salicylate derivatives on the silica-titania xerogel absorbance was studied. The equilibrium in these complexing reactions was shown to be reached in 15 min (Fig. 4). An earlier developed approach [23] allowed characterizing the heterogeneous reaction of salicylate derivatives complex forming. Half-reaction periods (T_1/2_) that characterize the reaction kinetics were calculated (4.5, 4.0, and 5.8 min for salicylate, salicylamide, and methylsalicylate, respectively).

The study of the surface complex formation process is an important step in the description of new solid materials properties [18, 19, 22, 24, 25]. The complex stoichiometry and the equilibrium constants were determined using the equilibrium shift method developed earlier by authors [18]. Solid phase spectrophotometric determination of salicylate derivatives using silica-titania xerogel was described by the following equation:

1 $$\equiv \overline{{{\text{Ti}} - ( {\text{OH)}}}}_{\text{n}} {\text{ + nHL }}=\, \equiv \overline{{{\text{Ti}} - {\text{L}}}}_{\text{n}} {\text{ + nH}}_{ 2} {\text{O}} . { }$$

The equilibrium constant of the reaction (1) can be described as following:

2 $${\text{K}}_{\text{eq}} { = [} \equiv \overline{{{\text{Ti}} - {\text{L}}}}_{\text{n}} ] / [\equiv \overline{{{\text{Ti}} - ( {\text{OH)}}}}_{\text{n}} ] [ {\text{HL]}}^{\text{n}} , { }$$

3 $${\text{lg([}} \equiv \overline{{{\text{Ti}} - {\text{L}}}}_{\text{n}} ] / [\equiv \overline{{{\text{Ti}} - ( {\text{OH)}}}}_{\text{n}} ] ) { } = {\text{ lgK}}_{\text{eq}} + {\text{ nlg[HL]}} . { }$$

$( [\equiv \overline{{{\text{Ti}} - {\text{L}}}}_{\text{n}} ] / [\equiv \overline{{{\text{Ti}} - ( {\text{OH)}}}}_{\text{n}} ] )$ can be defined as L, the rate of complexation of the titanium in the xerogel matrix ($[\equiv \overline{{{\text{Ti}} - {\text{L}}}}_{\text{n}} ]$ and $[\equiv \overline{{{\text{Ti}} - ( {\text{OH)}}}}_{\text{n}} ]$ are the concentrations of complexed and non-complexed titanium(IV) in the solid phase). L was calculated as A_i_/(A_ex_ − A_i_), where A_i_ is the xerogel’s absorbance after reaction and A_ex_ is the xerogel’s absorbance after reaction with the excess of corresponding salicylate derivative. Using the procedure described in [18] the lgL can be expressed as a linear function of lg[HL] [see Eq. (3)], which allows the determination of the complex stoichiometry, n, and the equilibrium constant, K_eq_. The equilibrium concentrations of salicylate derivatives in the liquid phase ([HL]) were determined using the preliminary constructed calibration curves and the absorbance of the salicylate derivatives solutions after the contact with the silica-titania xerogel powders. The data were approximated by linear dependences in the coordinates lgL on lg[HL] using the least squares method. The slopes of the resultant dependences allowed determining the stoichiometry of the produced complexes while the intercept with the ordinate axis provided the equilibrium constants of the heterogeneous reactions. These characteristics of the salicylate derivatives complexes with titanium(IV) are given in Table 1. Salicylic acid can form complexes with one or two titanium ions as described in [22, 25], and the complexes with two metal ions are less stable, which corresponds with our data (salicylate complexes are less stable than the other complexes).

**Table 1:** Characteristics of titanium(IV)—salicylate derivatives complexes in solid phase

Salicylate derivative (L)	Ti:L ratio	lgK_eq_ in solid phase
Salicylate	1:0.5	2.0
Salicylamide	1:1	2.9
Methylsalicylate	1:1	3.4

Selected conditions were applied to solid phase spectrophotometric determination of salicylate derivatives.

### Analytical application

In order to develop analytical procedures of salicylate derivatives determination we investigated the dependency between the silica-titania xerogels absorbance and the analyte concentration. The calibration curves were constructed using standard sample solutions at ten concentrations with least squares linear regression method. Analytical ranges and limits of detection (LOD) are given in Table 2. The intercept values did not differ significantly (n = 3, p > 0.05) from the blank (absorbance of uncolored xerogel).

**Table 2:** Analytical characteristics of solid phase spectrophotometric determination of salicylate derivatives (λ 410 nm, time of contact is 15 min)

	Analytical range (mM)	Linear approximation	R	Limit of detection (mM)
Sodium salicylate	0.1–5	A = 150·C	0.9968	0.02
Salicylamide	0.5–10	A = 86·C	0.9983	0.03
Methylsalicylate	0.05–4.7	A = 125·C	0.9976	0.01

#### Salicylate determination in biological liquids

The developed solid phase spectrophotometric procedures were applied to biological liquids analysis (Table 3). The recoveries show that the components of synthetic serum and human urine did not interfere the salicylate determination significantly, which allowed the use of the silica-titania xerogel for biological liquids analysis.

**Table 3:** Recovery test of solid phase spectrophotometric determination of salicylate in biological liquids (n = 3, P = 0.95)

Sample	Added (mM)	Found (mM)	RSD (%)	Recovery (%)
Synthetic serum^a^	0.5	0.49 ± 0.10	10.9	98.0
	1.0	0.97 ± 0.15	8.4	97.0
	1.5	1.42 ± 0.05	1.9	94.6
	2.0	2.09 ± 0.04	1.0	104.5
Urine^b^	0	0.46 ± 0.07	8.3	
	0.25	0.70 ± 0.09	7.2	96.0
	0.625	1.08 ± 0.07	3.5	99.2

^a^Synthetic serum composition is taken from [12]

^b^Collected 1 h after oral administration of 1000 mg of acetylsalicylic acid

The analytical range of the developed procedure made it possible to determine various salicylate levels in the serum samples: below the therapeutic dose (<0.5 mM), the therapeutic dose (0.5–1.5 mM), and overdose (2.0 mM).

The analysis of the real sample of human urine proved that the developed procedure allowed the determination of salicylate processed by the organism which is often required in the medical applications.

The concentrations found in biological liquids samples have shown good agreement with the results of Trinder salicylate test (Table 4). The suitability of the proposed procedures for biological liquids analysis has been proven. The proposed procedures can be applied for low-cost, fast salicylate analysis. Compared to classical Trinder test the solid phase spectrophotometric procedures can be characterized by faster determination, lower content of harmful acidic compounds, and better stability.

**Table 4:** Solid phase spectrophotometric determination of salicylate derivatives in real samples using the standard addition method (n = 3, P = 0.95)

Sample	Analyte	Found (independent method)	Found (proposed method)
Deep Heat cream^a^	Methylsalicylate	128 mg/g (DC)	123 ± 8 mg/g
Citramon tablets^b^	Acetylsalicylic acid	240 mg/tablet (DC)	220 ± 10 mg/tablet
Acetylsalicylic acid tablets Tatkhim	Acetylsalicylic acid	500 mg/tablet (DC)	500 ± 20 mg/tablet
Acetylsalicylic acid tablets Medisorb	Acetylsalicylic acid	500 mg/tablet (DC)	510 ± 20 mg/tablet
Synthetic serum^c^, containing 1 mM salicylate	Salicylate	1.03 ± 0.05 mM (TT)	0.97 ± 0.15 mM
Urine sample^d^	Salicylate	0.42 ± 0.01 mM (TT)	0.46 ± 0.07 mM

*DC* content declared by manufacturer

*TT* Trinder salicylate test

^a^Contains additional active substances: menthol 59.1 mg/g, eucalyptus oil 19.7 mg/g, turpentine oil 14.7 mg/g

^b^Contains additional active substances: paracetamol 180 mg/tablet, caffeine 30 mg/tablet

^c^Synthetic serum composition is taken from [12]

^d^Collected 1 h after oral administration of 1000 mg of acetylsalicylic acid

#### Determination of salicylate derivatives in pharmaceuticals

The determination of salicylate derivatives in pharmaceutical samples always comprises the difficulties of various interferences, as many drugs have complex composition and may contain other active substances in comparable quantities.

The difference in the pH optima for complex formation was used for salicylate/methylsalicylate determination in their mixtures (Table 5), which can also be important for mixed formulations.

**Table 5:** Determination of salicylate and methylsalicylate in mixed solutions (n = 3, P = 0.95)

Composition	pH	Found, mM
Salicylate 2.5 mM Methylsalicylate 3.75 mM	2.0	Salicylate 2.5 ± 0.2
Methylsalicylate 1.9 mM Salicylate 5.0 mM	7.6	Methylsalicylate 1.97 ± 0.5

The procedures for solid phase spectrophotometric determination of salicylate derivatives were applied to various pharmaceuticals analysis with the use of standard addition method. Acetylsalicylic acid was hydrolyzed to salicylate in the presence of sodium hydroxide prior to analysis. Reproducibility of real samples analysis is presented in Table 6. Relative standard deviation of 2–13 % and recovery of 90–100 % were achieved.

**Table 6:** Recovery test of solid phase spectrophotometric determination of salicylate derivatives (n = 3, P = 0.95)

Analyte	Sample	Added (mM)	Found (mM)	RSD (%)	Recovery (%)
Methylsalicylate	Deep heat cream^a^	0	1.01 ± 0.24	13.6	
		0.91	1.85 ± 0.27	8.2	92.3
		1.82	2.83 ± 0.24	4.9	100.0
Acetylsalicylic acid	Acetylsalicylic acid tablets Medisorb	0	0.57 ± 0.06	6.4	
		0.55	1.07 ± 0.06	3.4	90.9
		1.1	1.68 ± 0.06	2.2	100.9
	Acetylsalicylic acid tablets Tatkhim	0	0.69 ± 0.09	7.6	
		0.55	1.20 ± 0.09	4.5	92.7
		1.1	1.79 ± 0.09	2.9	100.0
	Citramon tablets^b^	0	0.49 ± 0.09	10.6	
		0.55	1.02 ± 0.15	7.7	96.4
		1.1	1.58 ± 0.15	5.8	99.1

^a^Contains additional active substances: menthol 59.1 mg/g, eucalyptus oil 19.7 mg/g, turpentine oil 14.7 mg/g

^b^Contains additional active substances: paracetamol 180 mg/tablet, caffeine 30 mg/tablet

The salicylate derivatives concentrations found in the pharmaceuticals have shown good agreement with the content declared by the manufacturer (Table 4). Among other advantages the determination in the presence of other active substances should be noted. The analgesic, anti-inflammatory pharmaceuticals based on salicylate derivatives often contain other active substances, such as paracetamol, caffeine, or menthol, and the absence of their interference broadens the range of possible applications.

The proposed procedures can be characterized as simple and fast and do not require complex labware or special storage conditions, and the analytical range is suitable for pharmaceuticals analysis. The silica-titania xerogel used as the sensor material is highly stable compared to other sensor materials.

## Experimental

### Reagents

The following reagents were purchased from Acros Organics: hydrochloric acid, sodium tetraborate, sodium salicylate, methylsalicylate, salicylamide, sodium hydrocarbonate, citric acid, sodium chloride, aminoacids, titanium(IV) tetraethoxyde, and tetraethyl orthosilicate. All the reagents were of analytical grade, titanium(IV) tetraethoxyde was of technical grade.

Stock solutions of salicylate and salicylamide were prepared with doubly distilled water. Stock solution of methylsalicylate was prepared with ethanol. Only freshly prepared solutions of these substances were used.

Trinder reagent was prepared as in [13]: 4.0 g of iron (III) nitrate nonahydrate and 5.0 g of trichloroacetic acid were dissolved in 100.0 mL of doubly distilled water.

### Instrumentations

Silica-titania xerogel was obtained by drying in Ethos microwave complex (Milestone, Italy).

Light absorbance of the xerogels water suspensions was measured using KFK-3 spectrophotometer (ZOMZ, Russia) and glass cuvettes (0.1 cm). Cuvette filled with non-colored xerogel water suspension was used as blank.

Light absorbance of the salicylate derivatives solutions was measured using KFK-3 spectrophotometer (ZOMZ, Russia) and quartz cuvettes (1.0 cm).

The pH value was measured using Expert-001 (Econix Expert, Russia) potentiometer with pH-sensitive electrode.

Surface area and porosity BET analysis was carried by using ASAP 2000 (Micromeritics, Norcross, GA, USA).

### Synthesis of silica-titania xerogel

Silica–titania xerogel was obtained using earlier developed procedures [18]: 20.0 mL of 0.05 M hydrochloric acid in 50 % ethanol solution was added to 10.0 mL of the precursors mixture (12.5 % titanium(IV) tetraethoxyde, 87.5 % tetraethoxysilane) while stirring. The wet gel was formed in the next 72 h. The wet gels were dried at 800 W microwave irradiation for 10 min to get dry xerogels.

The main characteristics of the xerogel: BET surface area is 540 m^2^/g, micropore volume is 0.14 cm^3^/g.

### Interaction of silica-titania xerogel with salicylate derivatives at different pH

0.10 g of silica-titania xerogel was added to 5.0 mL of solution, containing 2 mM salicylate, 3 mM salicylamide, or 2.5 mM methylsalicylate. pH of the solution was adjusted adding 0.01–2.0 mL of 0.5 M sulfuric acid, or 1.0 mL of phosphate buffer (pH 4–8), or 1.0 mL of borate buffer (pH 8–12). The obtained mixture was shaken for 15 min. Then the xerogels light absorbance was measured at 410 nm and pH of the solution was measured.

### Interaction of xerogel with salicylate derivatives kinetics studies

0.10 g of silica-titania xerogel was added to 5.0 mL of solution, containing 2 mM salicylate (pH 2), 3 mM salicylamide (pH 2), or 2.5 mM methylsalicylate (pH 7.6).The obtained mixture was shaken for 2–20 min. Then the xerogels light absorbance was measured at 410 nm.

### Determination of complexes composition and equilibrium constants

0.10 g of silica-titania xerogel was added to 5.0 mL of solution, containing 0.1–25 mM salicylate (pH 2), 0.5–10 mM salicylamide (pH 2), or 0.05–4.7 mM methylsalicylate (pH 7.6). The obtained mixture was shaken for 15 min. Then the xerogels light absorbance was measured at 410 nm. The solution absorbance was measured at 340 nm for salicylate, 320 nm for salicylamide, 315 nm for methylsalicylate. The concentration of unreacted salicylate derivative left in solution was determined using calibration curve at the corresponding wavelength.

### Calibration curves

0.10 g of silica-titania xerogel was added to 5.0 mL of solution, containing 0.1–5 mM salicylate (pH 2), 0.5–10 mM salicylamide (pH 2), or 0.05–4.7 mM methylsalicylate (pH 7.6). The obtained mixture was shaken for 15 min. Then the xerogels light absorbance was measured at 410 nm. Calibration curves were obtained using the least squares method.

### Determination of salicylate derivatives in synthetic serum

Synthetic serum was prepared as in [12]. 1.0 mL of Trinder reagent was added to 5.0 mL of synthetic serum containing 0.1–2 mM salicylate. After 30 min the colored solution absorbance was measured at 620 nm (l 1.0 cm).

0.10 g of silica-titania xerogel and 0.1 mL of 0.5 M sulfuric acid were added to 5.0 mL synthetic serum containing 0.1–2 mM salicylate, the mixture was shaken for 15 min. After that the xerogel light absorbance was measured.

### Determination of salicylate derivatives in human urine sample

The research was carried out according to the World Medical Association Declaration of Helsinki, and informed consent was obtained from the subject. The research was also approved by MSU Bioethics Committee [decision N 23-ch(3)]. Author of this work volunteered for the research: the healthy volunteer received a 1000 mg acetylsalicylic acid dose by oral administration. After 1 h the urine sample was collected.

1.0 mL of 0–3.1 mM salicylate was added to 1.0 mL of urine, and then 2.0 mL of Trinder reagent was added. After 30 min the colored solution absorbance was measured at 620 nm (l 1.0 cm).

2.5 mL of 0–1.3 mM salicylate was added to 2.5 mL of urine, and then 0.10 g of silica-titania xerogel and 0.1 mL of 0.5 M sulfuric acid were added. The mixture was shaken for 15 min and the xerogel light absorbance was measured.

### Determination of methylsalicylate in pharmaceutical samples

1.50 g of Deep Heat cream was diluted in ~20 mL of ethanol, and then boiled for 5 min. After cooling the solution was filtered to a 25.0 mL volumetric flask. Then the flask was diluted to the mark with ethanol. 0.1 mL of diluted sample was mixed with 3.75 mL of doubly distilled water, 1.0 mL of borate buffer (pH 8.5), 0.15 mL of standard methylsalicylate solution, and 0.10 g of xerogel, and then shaken for 15 min. After that the xerogel light absorbance was measured. Methylsalicylate concentration was determined using standard addition method.

### Determination of acetylsalicylic acid in pharmaceutical samples

Tablets, containing acetylsalicylic acid, were ground to powder, and an amount of powder, containing ~0.05 g of acetylsalicylic acid was weighed. 5.0 mL of 2 M NaOH was added to the powder, and then diluted with ~20 mL of doubly distilled water. The solution was heated for 5 min. After the cooling the solution was filtered to a 50.0 mL volumetric flask. Then the flask was diluted to the mark with doubly distilled water. This acetylsalicylic acid hydrolysis procedure completeness was verified by applying it to standard solutions of acetylsalicylic acid. 10.0 mL of hydrolyzed sample was mixed with 20.0 mL of standard salicylate solution, then pH was adjusted to 6.2, and then the solution was transferred to a 50.0 mL volumetric flask, which was diluted to the mark with doubly distilled water. 2.5 mL of diluted sample was mixed with 2.5 mL of doubly distilled water, 0.1 mL of 0.5 M sulfuric acid, and 0.10 g of silica-titania xerogel, and then shaken for 15 min. After that the xerogels light absorbance was measured. Acetylsalicylic acid concentration was determined using standard addition method.

## Conclusion

The reliable and simple method of salicylate derivatives determination based on the xerogel incorporated titanium(IV) complexes with salicylate derivatives has been proposed. In comparison with other methods of salicylate derivatives determination the proposed method key characteristics is its simplicity, whereas the analytical ranges are comparable with other methods [5, 6, 10, 11]. The procedures for solid phase spectrophotometric determination of salicylate derivatives in biological liquids and pharmaceuticals have been proposed. These new sensor materials are stable and ready to use and can be successfully applied to biological liquids and pharmaceuticals analysis.

## References

[1] Barnett HJM, Hirsh J, Mustard JF (1992). Acetylsalicylic acid: new uses for an old drug.

[2] Torriero AAJ, Luco JM, Sereno L, Raba J (2004). Voltammetric determination of salicylic acid in pharmaceuticals formulations of acetylsalicylic acid. Talanta.

[3] Wu AHB, McKay C, Broussard LA, Hoffman RS, Kwong TC, Moyer TP, Otten EM, Welch SL, Wax P (2003). National academy of clinical biochemistry laboratory medicine practice guidelines: recommendations for the use of laboratory tests to support poisoned patients who present to the emergency department. Clin Chem.

[4] Aukunuru JV, Kompella UB, Betageri GV (2000). Simultaneous high performance liquid chromatographic analysis of acetaminophen, salicylamide, phenyltoloxamine, and related products. J Liq Chrom Rel Technol.

[5] Shabir GA, Bradshaw TK (2011). Development and validation of a liquid chromatography method for the determination of methyl salicylate in a medicated cream formulation. Turk J Pharm Sci.

[6] Trinder P (1954). Rapid determination of salicylate in biological fluids. Biochem J.

[7] Pulgarin JAM, Molina AA (2002). Direct determination of salicylamide in serum by matrix isopotential synchronous fluorimetry. Talanta.

[8] Pulgarin JAM, Molina AA, Robles ISF (2011). Simultaneous determination of salicylic acid and salicylamide in biological fluids. Spectrochim Acta Part A.

[9] Abdolmohammad-Zadeh H, Kohansal S, Sadeghi GH (2011). Nickel–aluminum layered double hydroxide as a nanosorbent for selective solid-phase extraction and spectrofluorometric determination of salicylic acid in pharmaceutical and biological samples. Talanta.

[10] Carvalhal RF, Machad DS, Mendes RK, Almeida ALJ, Moreira NH, Piazetta MHO, Gobbi AL, Kubota LT (2010). Development of a disposable amperometric biosensor for salicylate based on a plastic electrochemical microcell. Biosen Bioelectron.

[11] Umasankar Y, Ramasamy RP (2013). Highly sensitive electrochemical detection of methylsalicylate using electroactive gold nanoparticles. Analyst.

[12] Shahrokhian S, Amini MK, Kia R, Tangestaninejad S (2000). Salicylate-selective electrodes based on Al(III)and Sn(IV) Salophens. Anal Chem.

[13] Radecka H, Grzybowska I, Radecki J, Jakubowski P (2007). Salicylate determination in human plasma by ISEs incorporating Mn(III)-porphyrine and Zn(II)-dipyrromethene. Analyt Lett.

[14] Isa IM, Sohaimi NM, Hashim N, Kamari A, Mohamed A, Ahmad M, Ghani SA, Suyanta (2013). Determination of salicylate ion by potentiometric membrane electrode based on zinc aluminium layered double hydroxides-4(2,4-dichlorophenoxy)butyrate nanocomposites. Int J Electrochem Sci.

[15] Shishkanova TV, Videnska K, Antonova SG, Krondak M, Fitl P, Kopecky D, Vrnata M, Kral V (2014). Application of polyaniline for potentiometric recognition of salicylate and its analogues. Electrochim Acta.

[16] Marczenko Z, Balcerzak M (2000). Separation, preconcentration and spectrophotometry in inorganic analysis.

[17] Morosanova EI (2012). Silica and silica–titania sol–gel materials: synthesis and analytical application. Talanta.

[18] Morosanova EI, Belyakov MV, Zolotov YA (2012). Silicon–titanium xerogels: synthesis and application to the determination of ascorbic acid and polyphenoles. J Anal Chem.

[19] Morosanova EI, Belyakov MV, Zolotov YA (2012). Silica–titania xerogels: solid phase spectrophotometric and field test determination of hydrogen peroxide in disinfectants J. Anal Chem.

[20] Morosanova MA, Morosanova EI, Anisimov DI, Zolotov YA (2015). Using silica-titania xerogels for solid phase spectrophotometric determination of fluoride in oral hygiene products. Curr Anal Chem.

[21] Gigant K, Rammal A, Henry M (2001). Synthesis and molecular structures of some new titanium(IV) aryloxides. J Am Chem Soc.

[22] Regazzoni AE, Mandelbaum P, Matsuyoshi M, Schiller S, Bilmes SA, Blesa MA (1998). Adsorption and photooxidation of salicylic acid on titanium dioxide: a surface complexation description. Langmuir.

[23] Morosanova EI, Velikorodnyi AA, Zolotov YA, Skornyakov VI (2000). Modifying silicic acid xerogels and accelerating heterogeneous reactions with their participation with the use of microwave radiation. J Anal Chem.

[24] Kholin Y, Zaitsev V (2008). Quantitative physicochemical analysis of equilibria on chemically modified silica surfaces. Pure Appl Chem.

[25] Moser J, Punchihewa S, Infelta PP, Graetzel M (1991). Surface complexation of colloidal semiconductors strongly enhances interfacial electron-transfer rates. Langmuir.

